# An Overview of Anti-Eukaryotic T6SS Effectors

**DOI:** 10.3389/fcimb.2020.584751

**Published:** 2020-10-19

**Authors:** Julia Monjarás Feria, Miguel A. Valvano

**Affiliations:** Wellcome-Wolfson Institute for Experimental Medicine, Queen's University Belfast, Belfast, United Kingdom

**Keywords:** bacterial pathogenesis, secretion system, T6SS, host manipulation, effector

## Abstract

The type VI secretion system (T6SS) is a transmembrane multiprotein nanomachine employed by many Gram-negative bacterial species to translocate, in a contact-dependent manner, effector proteins into adjacent prokaryotic or eukaryotic cells. Typically, the T6SS gene cluster encodes at least 13 conserved core components for the apparatus assembly and other less conserved accessory proteins and effectors. It functions as a contractile tail machine comprising a TssB/C sheath and an expelled puncturing device consisting of an Hcp tube topped by a spike complex of VgrG and PAAR proteins. Contraction of the sheath propels the tube out of the bacterial cell into a target cell and leads to the injection of toxic proteins. Different bacteria use the T6SS for specific roles according to the niche and versatility of the organism. Effectors are present both as cargo (by non-covalent interactions with one of the core components) or specialized domains (fused to structural components). Although several anti-prokaryotic effectors T6SSs have been studied, recent studies have led to a substantial increase in the number of characterized anti-eukaryotic effectors. Against eukaryotic cells, the T6SS is involved in modifying and manipulating diverse cellular processes that allows bacteria to colonize, survive and disseminate, including adhesion modification, stimulating internalization, cytoskeletal rearrangements and evasion of host innate immune responses.

## Introduction

Gram-negative bacteria depend on specific secretion systems, numbered Type I through Type VII, to transport proteins outside the cell for survival and fitness. It has been estimated that >25% of pathogenic and non-pathogenic proteobacteria encode between one and six Type VI secretion systems (T6SS) (Bingle et al., 2008; Boyer et al., 2009). The T6SS is a dynamic contractile protein nanomachine, evolutionarily related to bacteriophage tails, which delivers protein effectors in a contact-dependent manner into diverse cellular types, including other bacteria, fungi, and host eukaryotic cells. As reviewed elsewhere (Records, 2011; Basler, 2015; Cianfanelli et al., 2016b; Nguyen et al., 2018; Cherrak et al., 2019; Navarro-Garcia et al., 2019; Hernandez et al., 2020), the T6SS gene cluster encodes 13 core components for apparatus assembly. The system can be divided in three substructures, (i) a membrane complex (TssJLM) anchored to the inner membrane and associated to the outer membrane (Aschtgen et al., 2008; Ma et al., 2009b; Durand et al., 2015; Logger et al., 2016; Rapisarda et al., 2019; Yin et al., 2019), (ii) a baseplate complex assembled by a wedge (TssEFGK) (Brunet et al., 2015; Cherrak et al., 2018; Nazarov et al., 2018) and a spike (VgrG and, in some cases PAAR proteins) (Shneider et al., 2013; Brunet et al., 2015; Renault et al., 2018) and (iii) the dynamic tail complex that comprises the inner tube (Hcp) (Ballister et al., 2008; Brunet et al., 2014; Douzi et al., 2014) and the contractile sheath (TssBC) that wraps around the Hcp tube and propels the spike (Bonemann et al., 2009; Basler et al., 2012; Broms et al., 2013; Zhang et al., 2013; Kube et al., 2014).

The T6SS can translocate effector proteins in two modular ways: binding of an additional protein domain to structural components of the needle, Hcp, PAAR, or VgrG (specialized or evolved effectors) or by non-covalent direct or indirect interactions, via adaptor proteins, with any of the components of the needle (cargo effectors) (Shneider et al., 2013; Durand et al., 2014; Whitney et al., 2014; Alcoforado Diniz et al., 2015; Ma et al., 2017; Pissaridou et al., 2018). There are T6SS effector chaperone (TEC), or adaptor (Tap-1), proteins that are essential for toxin loading and delivery through binding to VgrG and effector proteins (Liang et al., 2015; Bondage et al., 2016; Flaugnatti et al., 2016; Jana and Salomon, 2019). TEC and Tap-1 proteins share a highly conserved domain of unknown function (DUF4123) and are not secreted; they exhibit a low pI values and are often genetically encoded upstream of their cognate effector genes or downstream of *vgrG* genes (Liang et al., 2015; Unterweger et al., 2015). Proteins containing DUF2169 domains are commonly found downstream of *vgrG* and upstream of DUF4150-containing effector genes and also serve as adaptor or chaperone in binding the N-terminal PAAR or PAAR-like domains of its cognate effector to the tip for translocation (Bondage et al., 2016; Santos et al., 2019). The DUF1795 containing proteins, namely Eag proteins, bind and stabilize the N-terminal PAAR-containing domains of their cognate effectors. Eag chaperone family members are frequently encoded adjacent to putative effectors with predicted transmembrane domains (Cianfanelli et al., 2016a; Quentin et al., 2018).

It has been reported that the T6SS mainly functions as a device for inter-bacterial competition to inject toxic antibacterial proteins into rival bacterial cells, thus modulating polymicrobial communities. More recently, the range of known functions of the T6SS has extended, including action against microbial fungi, biofilm formation and transport of ions. The T6SS also functions as a classical virulence factor by delivering toxins that allow bacteria to manipulate and subvert eukaryotic cells.

The T6SS toxins targeting eukaryotic cells are varied in biological and biochemical functions (Hachani et al., 2016). In general, different bacterial species use and adapt their T6SS for specific roles according to the host, niche or survival strategy of the organism and there is also considerable diversity in effector portfolio. In this review, we discuss and summarize the activity, target and mode of delivery of eukaryotic cell-targeting T6SS toxins important in pathogenicity, which interact and manipulate different components of the host cell. The effectors below revised are categorized accordingly to the bacterial species that encodes them.

## T6SS Eukaryotic Effectors

**Figure 1:**
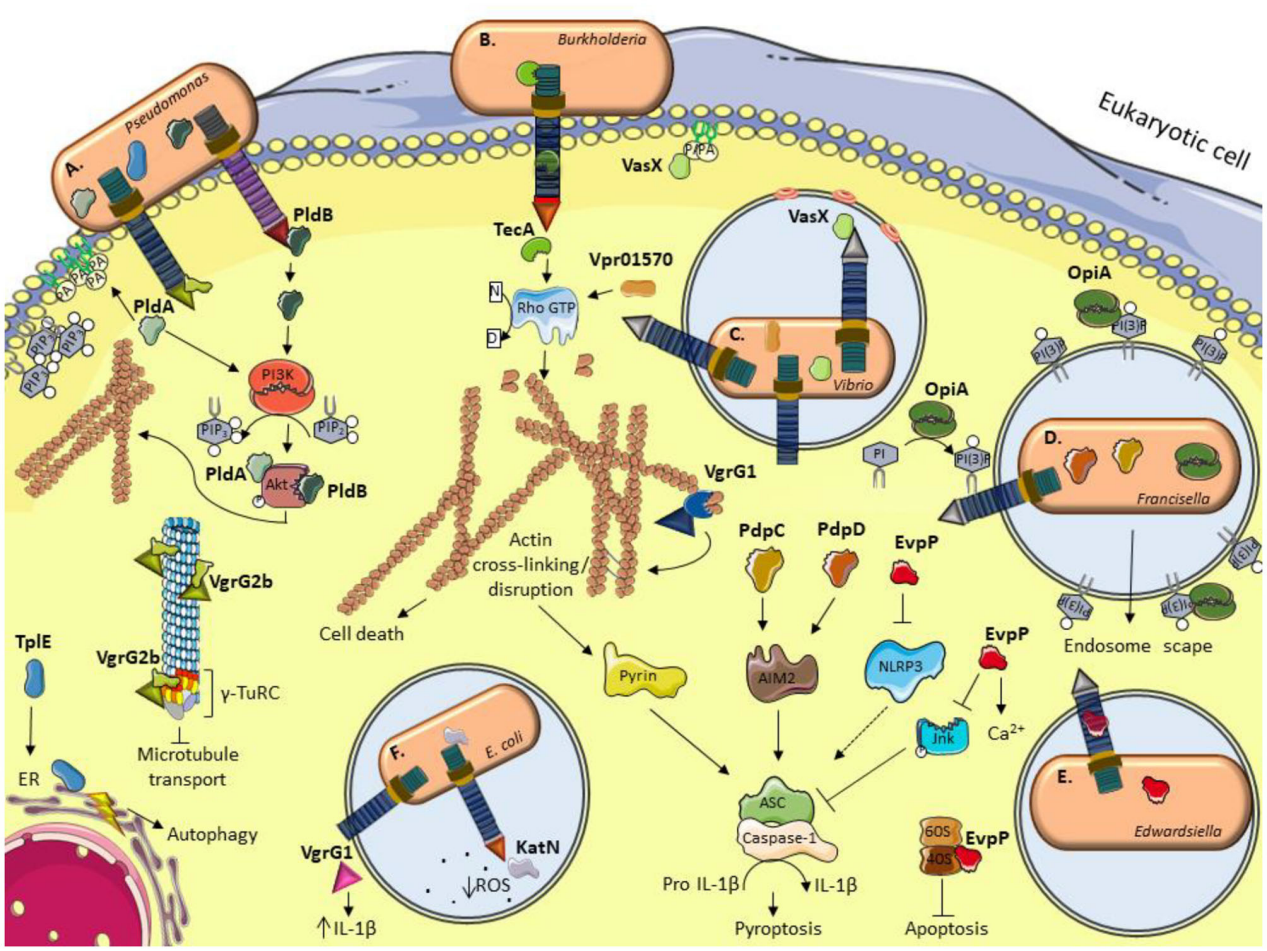
Schematic illustration of current models for the role of some anti-eukaryotic effectors. **(A)** *P. aeruginosa* delivers PldA and PldB which bind Akt to allow bacterial internalization via the induction of the PI3K pathway. VgrG2b enables bacterial internalization by interacting with members of the microtubule γ-TuRC complex. TplE targets the endoplasmic reticulum (ER) and induces stress and autophagy. **(B)** *B. cenocepacia* TecA is a deamidase that disrupts actin cytoskeleton by deamidating Rho GTPases and activates the Pyrin inflammasome. **(C)** The *V. cholerae* evolved VgrG1 interacts with and cross-link actin, leading to an accumulation of toxic actin oligomers and altering host cell morphology. VasX binds the lipid membrane phosphatidic acid (PA) and is thought to form pores in lipid bilayers. *V. proteolyticus* Vpr01570 contains a deamidase domain that activates Rho GTPases. **(D)** *F. tularensis* OpiA is a kinase able to phosphorylate phosphatidylinositol (PI) and binding to phosphatidylinositol trisphosphate [PI(3)P] for its recruitment to endosomal membranes. PdpC and PdpD activate AIM2 inflammasome. **(E)** *E. tarda* translocates EvpP which modifies calcium flux and has an inhibitory role in NLRP3 inflammasome by reducing Jnk phosphorylation and ASC oligomerization. *E. ictalurid* EvpP interacts with ribosomal protein S5 to negatively regulate apoptosis. **(F)** The EHEC effector KatN is a catalase that contributes to the survival in macrophages by hydrolyzing and decreasing the levels of reactive oxygen species (ROS). VgrG1 is secreted by ExPEC that alters the IL-1β levels. This figure was prepared using free templates on the Servier medical art website (https://smart.servier.com/).

Table 1 and Figure 1 give a general overview of functionalities of the eukaryotic T6SS effectors described in the text.

**Table 1:** List of anti-eukaryotic T6SS effectors and their functions.

**Organism**	**Effector**	**Function (biochemical activity)**	**References**
***Vibrio***			
*V. cholerae*	**VgrG1**	Contains an actin cross-linking domain (ACD) that binds and covalently cross-links actin, leading to an accumulation of toxic actin oligomers and altering host cell morphology, preventing host cell cytoskeletal rearrangements and disabling phagocytosis.	Pukatzki et al., 2007; Ma et al., 2009a; Ma and Mekalanos, 2010; Durand et al., 2012; Heisler et al., 2015; Dutta et al., 2019
	**VasX**	Required for virulence toward *Dictyostelium discoideum*. The PH domain binds host membrane lipids.	Miyata et al., 2011; Zheng et al., 2011; Dong et al., 2013
*V. proteolyticus*	**Vpr01580**	Predicted MIX-effector with a C-terminal domain homologous to cytotoxic proteins and other T6SS effectors that contain Rhs repeats.	Ray et al., 2017
	**Vpr01570**	MIX V effector containing a CNF1 domain that targets Rho GTPases resulting in actin cytoskeleton rearrangements in macrophages and toxicity to yeast.	Ray et al., 2017
	**Vpr00400**	Predicted effector homologous to the C-terminal domain of the insecticidal toxin Txp40 of *Xenorhabdus* and *Photorhabdus*.	Ray et al., 2017
***Escherichia coli***			
Enterohemorrhagic *E. coli*	**KatN**	Mn^2+^-containing catalase secreted into the host cell's cytosol after phagocytosis. It decreases the level of intracellular reactive oxygen species, enabling bacterial survival in macrophages.	Wan et al., 2017
Extra-intestinal pathogenic *E. coli*	**VgrG1**	Involved in bacterial adherence, multiplication, and evasion of innate immune responses.	Zong et al., 2019
***Pseudomonas***			
*P. aeruginosa*	**PldA**	Phospholipase D effector; it induces PI3K activation by interacting with Akt1 and Akt2 and promotes bacterial internalization into non-phagocytic cells.	Wilderman et al., 2001; Russell et al., 2013; Bleves et al., 2014; Jiang et al., 2014; Wettstadt et al., 2019
	**PldB**	Phospholipase D effector; it promotes bacterial internalization into epithelial cells *via* the induction of the PI3K/Akt pathway.	Bleves et al., 2014; Jiang et al., 2014
	**VgrG2b**	Enables entry into non-phagocytic cells by interacting with members of the microtubule γ-TuRC complex.	Sana et al., 2015; Wood et al., 2019
	**TplE**	Contains a eukaryotic PGAP1-like domain, which targets the host cell's ER leading to an unfolded protein response through the IRE1α-XBP1 pathway, which in turns induces stress and autophagy.	Jiang et al., 2016
***Klebsiella***			
*K. pneumoniae*	**Pld1**	Essential phospholipase for bacterial virulence in mice that plays a role in pathogenesis. It is encoded within a T6SS core gene cluster.	Lery et al., 2014
	**VgrG4**	Plays a role in T6SS-mediated intoxication of fungal cells.	Storey et al., 2020
***Francisella***			
*F. tularensis*	**PdpC**	Plays a role in phagosomal escape, trafficking to lysosomes, intramacrophage replication and is important for virulence *in vivo*. It is required for replication of bacteria in the liver and spleen of mice and for AIM2 inflammasome activation.	Lindgren et al., 2013a,b; Long et al., 2013; Uda et al., 2014; Eshraghi et al., 2016; Ozanic et al., 2016; Brodmann et al., 2017
	**PdpD**	Contributes to intramacrophage growth and phagosomal rupture. It is required to activate the AIM2 inflammasome.	Ludu et al., 2008; Eshraghi et al., 2016; Brodmann et al., 2017
	**OpiA**	Contributes to intramacrophage bacterial growth by promoting bacterial endosomal escape into the cytoplasm. It belongs to a family of bacterial PI3K enzymes and also plays a role in evasion of innate immunity in host cells by reducing the levels of TNF-α.	Eshraghi et al., 2016; Ledvina et al., 2018; Cantlay et al., 2020
	**OpiB**	Contributes to intracellular growth in phagocytic cells. The C-terminus is homologous to the ankyrin repeat domains and the N-terminus corresponds to an evolutionarily conserved cysteine protease.	Eshraghi et al., 2016
	**IglE**	It is translocated into macrophages and associates to microtubule organizing centers modulating membrane trafficking for bacterial intracellular growth.	Broms et al., 2012; Shimizu et al., 2019
***Edwardsiella***			
*E. tarda*	**EvpP**	The C-terminal domain interacts with EvpC and suppresses activation of the NLRP3 inflammasome by inhibiting the Ca^2+^-dependent MAPK-Jnk pathway. NLRP3 inhibition promotes bacterial colonization.	Zheng and Leung, 2007; Wang et al., 2009; Hu et al., 2014; Chen et al., 2017
*E. ictaluri*	**EvpP**	Plays a role in host cell colonization, apoptosis and necrosis in macrophages. Promotes adhesion and internalization.	Kalindamar et al., 2020
*E. piscicida*	**EvpP**	EvpP-inhibits the Jnk-MAPK pathway and Jnk-caspy inflammasome signaling pathways suppressing recruitment of neutrophils to infection sites and promoting bacterial colonization. Interacts with ribosomal protein S5 (RPS5) to regulate apoptosis.	Tan et al., 2019; Qin et al., 2020
***Burkholderia***			
*B. cenocepacia*	**TecA**	Disrupts macrophage actin cytoskeleton by deamidating Rho GTPases, which results in the activation of the Pyrin inflammasome.	Aubert et al., 2016
*B. pseudomallei and B. thailandensis*	**VgrG5**	The C-terminal domain is involved in mediating multinucleated giant cell formation, membrane fusion and virulence in mice.	Schwarz et al., 2014; Toesca et al., 2014
***Serratia***			
*S. marcescens*	**Tfe1**	Acts against fungal cells causing plasma membrane depolarization leading to cell death.	Trunk et al., 2018
	**Tfe2**	Acts against target fungal cells, leading to fungal cell death. Disrupts nutrient uptake and amino acid metabolism leading to the induction of autophagy.	Trunk et al., 2018
***Aeromonas***			
*A. hydrophila*	**VgrG1**	Targets the actin cytoskeleton. Has a vegetative insecticidal protein-2 domain with actin ADP-ribosyl transferase activity.	Suarez et al., 2010
***Yersinia***			
*Y. pseudotuberculosis*	**YezP**	Zn^2+^-binding effector that protects the pathogen from ROS and plays a role in virulence.	Wang et al., 2015

### Vibrio

*Vibrio cholerae* is a natural free-living bacterium widely distributed in aquatic environments and also the environment within human hosts. *V. cholerae* is a non-invasive intestinal pathogen; O1 and O139 serogroup cause the diarrheal disease cholera. The first reported T6SS toxin targeting eukaryotic cells was VgrG-1. Mutants unable to produce this protein lack the ability to secrete Hcp or to infect amoebae and mammalian macrophages, suggesting that rather in addition to be an essential component of the T6SS apparatus, VgrG-1 is a genuine effector (Pukatzki et al., 2007; Zheng et al., 2011). VgrG-1 carries a large (395 amino acids) C-terminal extension with homology to the actin cross-linking domain (ACD) of the RtxA toxin, a member of the MARTX family (Durand et al., 2012). VgrG-1 catalyzes *in vitro* the covalent cross-linking of two G-actin monomers in a Mg^2+^/Mn^2+^-ATP dependent manner and *in vivo* induced massive cross-linking of cytosolic actin in macrophages and from harvested intestines in an infant mice model of infection (Pukatzki et al., 2007; Ma and Mekalanos, 2010; Durand et al., 2012). Actin oligomers disrupt the normal inter-subunit interface in the actin filament and prevent polymerization (Satchell, 2009; Heisler et al., 2015). Bacterial internalization by endocytosis is needed for VgrG-1 ACD domain translocation into phagocytic cells to impair their function and cause cell death, preventing bacterial clearance from the gut (Ma et al., 2009a). VgrG-1 also possesses an actin binding motif (ABM) on the surface of the ACD similar to WH2 domain. Actin nucleation is inhibited by this ACD-ABM because the motif can bind and sequester actin monomers; this binding domain is also indispensable for ACD mediated actin cross-linking (Dutta et al., 2019). VgrG-1 forms homotrimeric and heterotrimeric complexes by interacting with VgrG-2 and VgrG-3 (Pukatzki et al., 2007). The crystal structure of VgrG-1-ACD (PDB 4DTD) reveals a V-shaped structure formed of 12 β-strands and 9 α-helices and an active site composed of 5 residues; one of these, Glu-16, is the critical residue for the cross-linking activity (Durand et al., 2012).

Another noteworthy T6SS *V. cholerae* protein is the virulence-associated secretion protein X (VasX or VCA0020), encoded in the T6SS gene cluster downstream of *hcp* and *vgrG-2*. The 121-kDa protein VasX requires the T6SS transcriptional activator VasH for expression, and a functional T6SS apparatus for secretion with the VgrG spike as carrier for its delivery. VasX plays a role in T6SS mediated virulence, killing amoebae by a mechanism that depends on actin cross-linking (Zheng et al., 2011; Dong et al., 2013). VasX carries an N-terminal Pleckstrin-homology (PH) domain that binds to membrane lipids including phosphatidic acid (PA) and each of the phosphatidylinositol phosphates (PIP). Since inositol phosphates are rarely found in bacteria the PH domain of VasX may have a role in binding to host membrane lipids and is thought to form pores in lipid bilayers (Miyata et al., 2011). VasX also carries a motif named MIX (marker for type six effectors) and a C-terminal colicin domain important for its secretion and T6SS assembly (Salomon et al., 2014; Liang et al., 2019).

*Vibrio proteolyticus* (Vpr) is a marine bacterium that has been previously isolated from corals with yellow band disease. Three T6SS effectors with putative anti-eukaryotic activities were identified by analyzing the Vpr secretome. Vpr01570 contains an N-terminal MIX V domain and a C-terminal CNF1 (cytotoxic necrotizing factor 1) deamidase domain that targets and activates Rho GTPases. Vpr01570 exogenously expressed in macrophages induces actin cytoskeleton rearrangements, including assembly of contractile actin stress fibers and ruffles at the top of the cells in a T6SS-dependent manner. Vpr01570 induces toxicity when expressed in yeast and these effects depend on the CNF1 domain (Ray et al., 2017). Vpr01580 is encoded next to the Vpr01570 encoding gene and also contains a MIX V domain; its homologous proteins are cytotoxic and contain Rhs repeats. Vpr00400 is homologous to the C-terminal domain of the toxic protein Txp40 which has insecticidal activity. Additional studies are required to elucidate the role of Vpr01580 and Vpr00400 (Ray et al., 2017).

### Escherichia coli

Enterohemorrhagic Escherichia coli (EHEC) is a human intestinal pathogen responsible for outbreaks of bloody diarrhea and hemolytic uremic syndrome worldwide. KatN is 84% identical to the Mn^2+^-containing catalase KatN of *Salmonella enterica* and the specific activity of KatN is 268.3 U/mg protein (Wan et al., 2017). KatN contributes to the EHEC response to oxidative stress *in vitro*; OxyR and RpoS are involved in *katN* transcription activation and H-NS, a global regulator, in its repression. After phagocytosis, EHEC induces the expression of T6SS, and translocated KatN contributes to the survival of intracellular bacteria in macrophages by hydrolyzing and decreasing the levels of reactive oxygen species (ROS) providing an ideal niche for bacterial growth and further infection (Wan et al., 2017).

Extra-intestinal pathogenic *Escherichia coli* (ExPEC) strains can cause urinary tract, bloodstream, prostate, and other infections at non-intestinal sites, leading to disease in humans and other animals. They are a serious threat to human public health and high risk for food safety. Porcine ExPEC causes meningitis, pneumonia, arthritis, and septicemia and is multidrug-resistant. The VgrG protein, a core component and a T6SS effector, performs diverse functions as an effector in addition to its structural component role. ExPEC VgrG1 plays a role in bacterial adherence, multiplication, and also a main role in evasion of innate immune response. In the absence of VgrG1, the serum level of IL-1β in mice is significantly reduced (Zong et al., 2019).

### Pseudomonas

One of the most virulent opportunistic pathogens is *Pseudomonas aeruginosa*, commonly found in soil and water as well as in plants and humans. *P. aeruginosa* is metabolically versatile and can cause a wide range of severe opportunistic infections in patients with cancer, cystic fibrosis and burns. The *P. aeruginosa* genome encodes three evolutionary distinct T6SS clusters, the H1–3-T6SSs, which are expressed simultaneously, each secreting a variable set of toxins. The H1-T6SS targets bacteria, while H2-3-T6SS targets bacteria and are also involved in internalization into eukaryotic cells (Mougous et al., 2006; Sana et al., 2012, 2016).

Phospholipases D (PLDs) are found in only a very limited number of prokaryotic organisms but, when present, they often play a role in bacterial pathogenesis. The 122-kDa protein PldA (Tle5a) from *P. aeruginosa* has high homology with eukaryotic PLDs; the protein is secreted via H2-T6SS and delivered as a cargo effector via their cognate VgrG4b. PldA possesses two HXKXXXXD catalytic motifs and it has phospholipase calcium-regulated activity *in vitro*. PldA enzymatic activity resulting in phosphatidylcholine hydrolysis depends on a catalytic histidine residue (H855) (Wilderman et al., 2001; Russell et al., 2013; Wettstadt et al., 2019). PldA can induce cell death through PA accumulation via PLD activity, primarily aimed against phosphatidylethanolamine (Russell et al., 2013; Jiang et al., 2014).

The 83-kDa protein PldB (Tle5b) is a *P. aeruginosa* H2- and H3-T6SS-dependent PLD effector delivered via their cognate VgrG5 and is able to translocate into human epithelial cells. PldB possesses two HXKXXXXD catalytic motifs that play a crucial role in toxicity. PldA and PldB do not share homology, suggesting that they have developed similar functions by convergent evolution (Jiang et al., 2014; Wettstadt et al., 2019). A study deciphering the prevalence of genes encoding T6SS effectors in clinical isolates found that the prevalence of *pldA* was increased in isolates responsible for severe acute pulmonary infection and septicemia. In contrast, *pldB* prevalence was high in all isolates (Boulant et al., 2018). PldA and PldB are not involved in bacterial adhesion but promote intracellular invasion of host eukaryotic cells by activation of the phosphatidylinositol 3-kinase (PI3K)/Akt signaling pathway that is crucial for cell growth, proliferation, and programmed cell death. After injection into epithelial cells, PldA and PldB directly interact with Akt1 and/or Akt2 kinase, resulting in activation of the PI3K-Akt pathway. Indeed, Akt phosphorylation at serine 473 promotes remodeling of the apical membrane in which protrusions enriched in phosphatidylinositol-3,4,5-triphosphate (PIP_3_) and actin enables bacterial entry (Bleves et al., 2014; Jiang et al., 2014).

VgrG2b is conserved in all *P. aeruginosa* strains present in the *Pseudomonas* genome database. VgrG2b is a 113-kDa protein that contains the conserved VgrG domain homologous to gp27 and gp5 phage-tail proteins followed by a domain of unknown function, DUF2345, and a C-terminal extension with a Zn^2+^-dependent metallopeptidase domain (LFIHEMTHVW). It is an evolved VgrG with double function as a structural component of the secretion machinery and a true effector translocated via the H2-T6SS required for invasion of host cells. VgrG2b injection precedes internalization; its C-terminal domain interacts with α- and β-tubulin complexes and with the γ-tubulin complexes, such as the γ-tubulin small complex (γTuSC) and the γ-tubulin ring complex (γ-TuRC) involved in microtubule nucleation. This interaction allows bacterial uptake into epithelial cells to be mediated by actin cytoskeletal rearrangement (Sana et al., 2015; Wood et al., 2019). The crystal structure of Vgr2b C-terminal encompassing residues 833-1019 (PDB 6H56) presents a metallopeptidase fold (Wood et al., 2019).

Sana et al. (2016) proposed a working model for the interplay of T6SS effectors PldA, PldB and VgrG2b in *P. aeruginosa* internalization. First, VgrG2b is translocated via H2-T6SS, causing the polarization of epithelial cells by targeting the microtubule network, promoting microtubule nucleation at the membrane by interacting with γ-TuRC. These novel sites of non-radial microtubule nucleation interfere with the transport of microtubule-dependent cargoes in the cell, like PI3K. Simultaneously, PldA and PldB are translocated by the different H2 and H3-T6SSs, activating Akt which allows actin-dependent membrane protrusion that enables bacterial internalization into the epithelial cells.

Another effector, TplE, contains a eukaryotic PGAP1 (post-glycosylphosphatidylinositol attachment to proteins 1)-like domain. TplE is translocated into epithelial cells in an H2-T6SS-dependent manner and localizes to host endoplasmic reticulum (ER), causing a contraction of the ER surrounding the nuclear periphery. TplE phospholipase activity is not involved in localization but is required for disruption of ER structure. TplE induces the upregulation of Bip and CHOP chaperones that are biomarkers for ER stress and induces the splicing of XBP1 mRNA, suggesting that the TplE-induced unfolded protein response is dependent on the IRE1α-XBP1 signaling pathway. It was also reported that autophagic flux is induced by TplE delivery into human epithelial cells (Jiang et al., 2016).

### Klebsiella

*Klebsiella pneumoniae* is a ubiquitous species in nature, a gut commensal, and an opportunistic pathogen in humans. As a prominent nosocomial pathogen, it can cause a wide range of infections, including urinary tract, respiratory tract or blood infections, bacteremia and liver abscesses. Due to the regular occurrence of multiple antibiotic-resistant isolates, *K. pneumoniae* is considered a global public health concern. In *K. pneumoniae* three different T6SS loci were defined, and a gene encoding a PLD family protein Pld1 is located within a type VI secretion system locus (Sarris et al., 2011; Lery et al., 2014). Pld1 is a Tle5 homolog, has two conserved HXKXXXXD motif and is expressed during *K. pneumoniae* virulence in a mouse model of pneumonia. The *pld1* phospholipase mutant was strongly attenuated *in vivo*, suggesting an effect on lipid metabolism in *K. pneumoniae* pathogenesis (Lery et al., 2014).

VgrG4 encodes a C-terminal domain of unknown function DUF2345. VgrG4 is needed for bacteria-induced killing of the fungal pathogen *Candida albicans* and *Saccharomyces cerevisiae*, implicating the T6SS in intoxication of fungal cells. The DUF2345 domain is sufficient for the anti-eukaryotic activity (Storey et al., 2020).

### Francisella

*Francisella tularensis* is one of the most infectious intracellular pathogens known. After entering the body via the skin, mucous membranes, or respiratory or gastrointestinal tracts, it causes tularemia, a necrotizing bronchopneumonia that leads to sepsis and death. The T6SS encoded by the *Francisella* pathogenicity island (FPI) is critical for the virulence of this bacterium. In contrast *F. tularensis* subsp. *novicida* (*F. novicida*) has low virulence in humans, but is highly virulent in mice and thus often used as a laboratory model for tularemia (Eshraghi et al., 2016). PdpC (pathogenicity determinant protein C) is a 156-kDa protein encoded within the FPI that contributes to phagosomal escape, trafficking to lysosomes and intramacrophage replication. PdpC plays a role in virulence in the mouse model, as demonstrated by the Δ*pdpC* mutant causing significantly lower mortality in mice with a corresponding reduction in bacterial burden in organs. PdpC is required to activate the AIM2 inflammasome and Δ*pdpC* induces lower levels of type I interferon production (Lindgren et al., 2013a,b; Long et al., 2013; Uda et al., 2014; Eshraghi et al., 2016; Ozanic et al., 2016; Brodmann et al., 2017).

PdpD is a protein encoded within the FPI; its export requires VgrG and PdpA. This effector contributes to intramacrophage growth and phagosome rupture. PdpD is also required to activate the AIM2 inflammasome (Eshraghi et al., 2016; Brodmann et al., 2017).

OpiA and OpiB are encoded by open reading frames located outside of the FPI and recently identified as T6SS substrates. They contribute to intracellular growth. There are no homologs of OpiA found outside of *Francisella*, and *in silico* analyses were unable to identify characterized domains or motifs within the protein. The OpiB C-terminus is homologous to the ankyrin repeat domains mediating protein-protein interactions that are normally found in eukaryotic proteins. The OpiB N-terminus constitutes an evolutionarily plastic cysteine protease (Eshraghi et al., 2016). OpiA belongs to a family of wortmannin-resistant bacterial PI3K enzymes with members found in a wide range of intracellular pathogens. OpiA can phosphorylate PI but not PIP_2_. OpiA binds phosphatidylinositol 3-phosphate [PI(3)P] in a selective and high-affinity manner serving as a mechanism for the specific recruitment of OpiA to endosomal membranes. OpiA acts on the *Francisell*a-containing phagosome, leading to efficient bacterial escape from late endosomes into the cytoplasm of infected cells (Ledvina et al., 2018). The protein is translocated into phagocytic cells and reduces the levels of TNF-α, a pro-inflammatory cytokine from monocytes required to block intracellular replication. OpiA contributes to the pathogenesis of *F. tularensis*, as demonstrated using a chicken embryo infection model (Cantlay et al., 2020).

The protein IglE (intracellular growth locus E) is translocated into macrophages (Broms et al., 2012). The Δ*iglE* mutant has a slower intracellular growth rate in human macrophages, suggesting a role for this protein in intracellular replication. IglE interacts with β-tubulin, pericentrin and with microtubule organizing centers. It inhibits the dynein- based intracellular trafficking in host cells, allowing *F. novicida* to escape from fusion with lysosomes (Shimizu et al., 2019).

### Edwardsiella

*Edwardsiella tarda* infects a wide range of hosts including fish, birds, reptiles and humans. In humans, it causes both intestinal and extra-intestinal infections, mainly in individuals with impaired immune systems. Edwardsiellosis in fish is a devastating disease predominant in worldwide aquaculture industries, making it of particular importance to the fishing industry (Zheng and Leung, 2007). EvpP (*E. tarda* virulence protein P) transcription is iron-dependent. EvpP is a 20-kDa protein that is not conserved in other bacteria and contains no conserved domains or motifs. It is secreted via T6SS and the EvpP C-terminus interacts with EvpV (Hcp homolog) (Zheng and Leung, 2007; Hu et al., 2014). In an *in vivo* fish model, EvpP plays a role in proliferation and infection. This toxin also mediates hemolytic activity in sheep erythrocytes and contributes to mucus adhesion and serum resistance of Japanese flounder. EvpP is important for internalization into epithelial papilloma of carp cells (Wang et al., 2009). The protein localizes in the membrane after injection and has an inhibitory role in NLRP3 inflammasome activation by reducing Jnk phosphorylation and ASC oligomerization. It was reported that Δ*evpP* induced higher intracellular calcium flux than wildtype *E. tarda* indicating that EvpP-mediated manipulation of the Jnk-ASC could be traced upstream to intracellular Ca^2+^ signaling (Chen et al., 2017).

*Edwardsiella ictaluri* causes enteric septicemia of catfish and is the most important endemic infectious disease in catfish aquaculture industry. EvpP toxin is involved in adhesion and internalization of *E. ictaluri* in catfish ovary cells. EvpP plays a role in growth regulation in the phagolysosome where oxidative stress and limited nutrients are present, and also favors survival and increases apoptosis and necrosis in catfish anterior kidney macrophages (Kalindamar et al., 2020).

*Edwardsiella piscicida* is abundant in water and causes food and waterborne infections in fish, animals and humans (Leung et al., 2019). Using an *in vivo* zebrafish larvae infection model EvpP inhibits immune cells recruitment via Jnk-MAPK signaling cascades. EvpP reduces the expression of *cxcl8a* (chemokine ligand 8) and *mmp13* (matrix metallopeptidase 13) transcripts, indicating that EvpP plays a role in inhibiting the recruitment of neutrophils. Meanwhile, EvpP also inhibits the Jnk-caspy inflammasome and IL-1β expression to suppress neutrophil recruitment, thereby promoting bacterial colonization (Tan et al., 2019). EvpP is also able to reduce Annexin V binding and activation of cleaved caspase-3 involved in apoptosis. This effector interacts with ribosomal protein S5 (RPS5), most likely resulting in downregulation of apoptosis-associated pathways in macrophages (Qin et al., 2020).

### Burkholderia

*Burkholderia cenocepacia* is widespread in the environment, particularly within the rhizosphere. *B. cenocepacia* is also an opportunistic pathogen causing chronic lung infections in patients with cystic fibrosis as well as in other immunocompromised patients (Loutet and Valvano, 2010). The 17-kDa protein TecA is a non-VgrG T6SS effector responsible for actin disruption *in vivo*. TecA and other bacterial homologs bear a cysteine protease-like catalytic triad, which inactivates Rho GTPases by deamidating a conserved asparagine in the GTPase switch-I region. RhoA deamidation induces Pyrin inflammasome activation (Aubert et al., 2016).

*Burkholderia thailandensis* is a soil saprophyte of low virulence. *Burkholderia pseudomallei* is the causative agent of melioidosis, a serious and often fatal human infection. These species, referred as the Bptm group, encode several T6SSs but the type VI secretion system 5 (T6SS-5) is the one required for virulence in mammalian infection models. VgrG-5 is a substrate of T6SS-5 and is translocated into macrophages. VgrG-5 C-terminal domain is involved in mediating multinucleated giant cell formation, membrane fusion and virulence in mice (Schwarz et al., 2014; Toesca et al., 2014).

### Serratia

*Serratia marcescens* occurs naturally in soil and water. It is associated with urinary and respiratory infections, endocarditis, osteomyelitis, septicemia, wound infections, eye infections, and meningitis. Tfe1 (T6SS antifungal effector 1) is an antifungal small T6SS toxin (20 kDa), deletion of Tfe1 encoding gene resulted in a four-fold increase in recovery of viable *Candida albicans* target cells compared with the wild type bacteria. Tfe1 causes cell distortion and lysis in both the budding and filamentous forms of *C. albicans*. Tfe1 inhibits growth of *S. cerevisiae* and induces abnormally large vacuoles and cell lysis, confirming the fungicidal role of this effector. Tfe1 intoxication results in membrane depolarization by loss of membrane potential, which is not due to pore formation but can lead to a loss of membrane integrity and cell death (Trunk et al., 2018, 2019). Removal of Tfe2 (T6SS antifungal effector 2) encoding gene, resulted in almost complete loss of activity against *S. cerevisiae* or *Candida glabrata* and reduced activity against *C. albicans*. Tfe2 is a small protein (26 kDa) which, when expressed in *S. cerevisiae*, is able to inhibit its growth. Tfe2 intoxication disrupts nutrient uptake and amino acid metabolism and causes autophagy. Tfe1 and Tfe2 act on different cellular targets in fungal cells (Trunk et al., 2018, 2019).

### Aeromonas

*Aeromonas hydrophila* is common in freshwater environments and causes disease in fish, reptiles, amphibians, and humans. It causes a broad spectrum of infections (including septicemia, meningitis, endocarditis) in humans and severe motile septicemia in warmwater fishes. The 103-kDa protein VgrG1 is translocated by the T6SS. VgrG1 contains a vegetative insecticidal protein domain at its C-terminus with actin ADP-ribosyltransferase activity. This effector alters the actin cytoskeleton and induces apoptosis in epithelial cells (Suarez et al., 2010).

### Yersinia

*Yersinia pseudotuberculosis* is an enteric pathogen, which usually grows in the environment and can be transmitted to mammalian hosts through ingestion of contaminated food or water. It typically causes a broad range of gastrointestinal diseases, from enteritis to mesenteric lymphadenitis (Yang et al., 2018). *Y. pseudotuberculosis* contains four T6SS clusters. The T6SS-4 secreted substrate YezP (*Yersinia* extracellular zinc-binding protein) is a Zn^2+^-binding protein that has the ability to rescue the sensitivity to oxidative stress exhibited by T6SS mutants when added to extracellular milieu. YezP plays a role in virulence for mice but its contribution to the infection process requires additional investigation (Wang et al., 2015).

## Concluding Remarks

Bacterial pathogens employ many strategies to invade mammalian hosts, damage tissues, organelles and prevent the immune system from responding. One strategy is the secretion of proteins (effectors) across membranes. As we described in this review, these toxins are secreted and injected into host cells via the T6SS and exist both as evolved VgrGs and cargo effectors. Translocated effectors can play many roles in eukaryotic cells, which promote bacterial virulence ranging from attachment to directly intoxicating target cells and disrupting their functions to finally establishing a replicative niche and successful colonization.

The clearance of pathogens depends on the host innate immune responses that take place at early stages of infection and in which macrophages and neutrophils are the essential players. Once inside the macrophage, intracellular bacteria can reside in vacuoles or in the cytosol, depending on their effector repertoire which help them to evade host defense and continue the infection cycle and replicate. Here, we described 27 T6SS effectors employed by several bacterial species to promote virulence in eukaryotic cells. These effectors can display similar or complementary functions into host cells and modulate the same central pathway of the host cell (e.g., inflammasome) or having different roles. Moreover, a pathogen may secrete several proteins to produce the same outcome (e.g., PldA and PldB).

*Vibrio, Pseudomonas, Burkholderia* and *Aeromonas* species translocate toxins in a T6SS-dependent manner leading to resistance to phagocytosis, inflammasome activation, as well as bacterial internalization by manipulating the actin cytoskeleton. *Vibrio, Pseudomonas*, and *Burkholderia* in particular, disrupt the host cell cytoskeleton, targeting actin, although *Pseudomonas* and *Francisella* effectors target the microtubules. The interference with immunity pathways is a hallmark function achieved by T6SS-dependent effectors. *Burkholderia, Vibrio, Francisella* and *Edwardsiella* inject toxins involved in the activation of the different inflammasomes that lead to the secretion of proinflammatory cytokines. In this context, activation of the inflammasome can be important for the clearance of the pathogen, suggesting the possibility that the T6SS effectors may also have a role as anti-virulence factors. This notion is supported from results using TecA deficient mutants in experimental mice infection whereby the mutant bacteria were able to kill infected mice while the parental strain was cleared. This clearance effect was abolished in infections using Pyrin inflammasome-defective mice (Aubert et al., 2016).

Another important mechanism of host defense is the generation of reactive oxygen species to eradicate intracellular bacteria. *E. coli* and *Yersinia* T6SSs deliver effectors with the ability to modulate the oxidative stress and protect the pathogen from ROS and allowing growth. Finally, *Serratia* delivers effectors into fungal cells, causing depolarization of the plasma membrane and metabolism disrupted, leading to cell death.

In recent years, remarkable progress has been made toward elucidating the function of eukaryotic effectors of the T6SS, which has contributed to better understand several aspects of bacterial pathogenesis. However, our understanding of the molecular mechanism of many T6SS-secreted toxins awaits detailed functional analysis, including biochemical, biophysical, immunological and structural studies. The kinetics of effector delivery is also an open question since very little is known on whether their translocation is regulated in a temporal and spatial manner and the signals that triggers their secretion.

## Author Contributions

JM and MV: wrote the manuscript.

## Conflict of Interest

The authors declare that the research was conducted in the absence of any commercial or financial relationships that could be construed as a potential conflict of interest.

## Figures and Tables

**Table:** 

ABM	actin binding motif
ACD	actin cross-linking domain
CNF1	cytotoxic necrotizing factor 1
EHEC	enterohemorrhagic *Escherichia coli*
ER	endoplasmic reticulum
ExPEC	extra-intestinal pathogenic *Escherichia coli*
FPI	Francisella pathogenicity island
MIX	marker for type six effectors
PA	phosphatidic acid
PH	Pleckstrin-homology
PI	phosphatidylinositol
PI(3)P	phosphatidylinositol 3-phosphate
PI3K	phosphatidylinositol 3-kinase
PIP	phosphatidylinositol phosphates
PIP_3_	phosphatidylinositol-3,4,5-triphosphate
PLD	phospholipase D
ROS	reactive oxygen species
γ-TuRC	gamma-tubulin ring complex
